# When to Operate on Pediatric Patients with Congenital Heart Disease and Pulmonary Hypertension

**DOI:** 10.5935/abc.20170134

**Published:** 2017-09

**Authors:** Antonio Augusto Lopes, Ana Maria Thomaz

**Affiliations:** Instituto do Coração (InCor) - Faculdade de Medicina - Universidade de São Paulo, São Paulo, SP - Brazil

**Keywords:** Heart Defects, Congenital/surgery, Heart Defects/complications, Hypertension, Pulmonary/congenital, Postoperative Care/complications

In pediatric patients with congenital heart disease associated with pulmonary arterial hypertension (PAH-CHD), deciding about surgery may be difficult depending on the diagnostic scenario. Most patients with communications between the cardiac chambers or the great arteries can now be operated on quite safely, with excellent results. Pulmonary hypertension accounts for complications in less than 10% of cases. In general, it has been considered that early assignment to surgery is the best strategy to avoid complications. This is unquestionable. However, late referral is still a problem in developing countries and underserved areas. Furthermore, it must be acknowledged that severe pulmonary vasculopathy may be present early in life, leading to the speculation that vascular lesions may develop from birth, or even before. Moderate to severe pulmonary vascular abnormalities limit the success of the repair of cardiac anomalies. First, the so-called postoperative pulmonary hypertensive crises are relatively infrequent in the present era, but still associated with high mortality rates (>20%).^1^ Patient management requires sophisticated armamentarium for life support, sometimes extracorporeal membrane oxygenation (ECMO). Second, patients surviving the immediate postoperative period may remain at risk of persistent postoperative PAH, which is associated with poor outcome compared to other etiologies of pediatric PAH.^2^ Therefore, while becoming aware of these complications, clinicians and surgeons need to get together and plan the best therapeutic strategy on an individual basis.

For a long time, cardiac catheterization (with the acute pulmonary vasodilation test, AVT) has been considered as a gold-standard assessment of PAH-CHD. In most tertiary centers indeed, catheterization data occupy a high hierarchical position in the decision to operate on PAH-CHD patients. Also, subclassifications of PAH-CHD according to disease severity are based on hemodynamic parameters. However, obtainment and interpretation of catheterization data are not easy tasks, especially in the pediatric population, for several reasons:

1- the procedure is generally performed under general anesthesia, mechanical ventilation and muscle relaxation, therefore, far from the physiological conditions;
2- even mild systemic hypotension (for example, due to inadequate hydration in regard to the effects of anesthetic drugs) makes results impossible to analyze in subjects with systemic-to-pulmonary shunts;
3- direct measurement of oxygen consumption, which is essential for calculations of pulmonary and systemic blood flow, is not done in many institutions;
4- inhaled nitric oxide is expensive and, therefore, unavailable in many centers, limiting the performance of the AVT; it is widely known that challenging the pulmonary circulation with ~100% oxygen is not adequate to test for vasoreactivity, leading to inaccurate results; and
5- there has been no consensus about the protocol for the AVT in the pediatric population, and the magnitude of the response does not correlate with outcomes in CHD.^3^ In view of all these difficulties, cardiac catheterization remains as an important step in the evaluation of PAH-CHD,^4^ but data are now taken into consideration as part of the whole diagnostic scenario.

In the era of the so-called specific drugs for the management of PAH, there have been attempts to treat inoperable patients (older subjects with elevated pulmonary vascular resistance and sometimes bidirectional shunting across the communications) aiming at making them operable. This approach has been referred to as “treat-and-repair strategy”. However, there has not been sufficient evidence to support such recommendation in a generalized way.^5^ On one hand, there is no guarantee that drugs will remain effective over the long term. On the other, persistence of severe PAH is a stormy complication after repair of congenital cardiac shunts, with significantly reduced survival.^2^ Reopening of the communication frequently requires reoperation under cardiopulmonary bypass, a high-risk procedure in PAH patients. In selected cases, repairing an extracardiac lesion while leaving an intracardiac communication unrepaired, or considering partial closure of the defect may be an option.

Choosing the best therapeutic strategy in PAH-CHD, especially in the pediatric population, is something to be done on an individual basis. Sometimes, surgery must be considered even without expectation of complete hemodynamic normalization. This may be the case, for example, of a child with unrestrictive ventricular septal defect and PAH, with severe mitral regurgitation and nearly failing left ventricle. In this case, left heart disease will probably be more life limiting than PAH itself. Therefore, one could attempt to define operability in a general sense, not linking it to any single specific index of parameter cut-off. A patient should be deemed operable, if on the basis of all diagnostic data, the multiprofessional team is convinced that surgery can be offered with acceptable risk, with significant benefits envisioned over the medium and long term.

We would like to complement this view on the problem by presenting a summary of clinical features and diagnostic parameters that have been used for decision making about surgery in PAH-CHD, with emphasis on the pediatric population (Table 1). Age and complexity of the cardiac anomaly must be considered. For example, while *truncus arteriosus* is now successfully repaired early in life, it is associated with development of severe pulmonary vasculopathy with increasing age. Echocardiography is useful for assessing the severity of PAH and right ventricular adaptation or dysfunction, provided that numeric parameters can be obtained in addition to anatomic information. Echocardiography is particularly useful when repeated measurements are needed in pediatric patients, before and after operation. Finally, cardiac catheterization with direct measurement of pulmonary vascular resistance should be considered in all patients with unrestrictive cardiac septal defects with no history of congestive heart failure and failure to thrive. Rather than looking at a single parameter, the best policy is to use a holistic diagnostic approach in these delicate patients.^6^ In terms of decision making about surgery, “benign neglect” is probably the best humanistic attitude when risk overcomes benefits. Otherwise, decision to operate must be based on multiple diagnostic aspects and the opinion of an expert multiprofessional team.

**Table 1:** Clinical features and noninvasive and invasive parameters considered for decision making about surgery in pediatric PAH-CHD

Favorable scenario	Parameters	Unfavorable scenario
< 9 months	Age	> 2 years
Pre-tricuspid or simple post-tricuspid defects	Complexity of cardiac anomaly	Complex defects Truncus arteriosus Transposition of the great arteries Atrioventricular septal defect in Down syndrome
Present in clinical history / physical examination	Congestive heart failure Pulmonary congestion Failure to thrive	Absent
> 93%, no gradient	Systemic oxygen saturation, gradient right arm vs. lower extremities	< 90%, presence of gradient
	**Echocardiographic parameters**	
> 2.5	Pulmonary-to-systemic blood flow ratio (Qp/Qs)	< 2.0
> 24 cm	Velocity-time integral of blood flow in pulmonary veins	< 20 cm
Left to right	Direction of flow across the cardiac communication	Bidirectional
	**Cardiac catheterization**	
≥ 20%	% decrease in pulmonary-to-systemic vascular resistance ratio (Rp/Rs) from baseline, during nitric oxide (NO) inhalation	< 20%
< 5.0 Wood units•m^2^	Level of pulmonary vascular resistance achieved on NO	> 8.0 Wood units•m^2^
< 0.27	Lowest Rp/Rs achieved during NO inhalation	> 0.33
